# Golfers have greater preoperative and equal postoperative function when undergoing total knee arthroplasty compared to non-golfers

**DOI:** 10.1007/s00590-022-03253-8

**Published:** 2022-04-01

**Authors:** P. G. Robinson, R. S. Kay, D. MacDonald, A. D. Murray, N. D. Clement

**Affiliations:** 1 Edinburgh Orthopaedics, Royal Infirmary of Edinburgh, Edinburgh, UK; 2 PGA European Tour Performance Institute, Virginia Water, UK; 3 Edinburgh Medical School, University of Edinburgh, Edinburgh, UK; 4 Sports and Exercise Medicine, University of Edinburgh, Edinburgh, UK; 5 Medical and Scientific Department, The R&A, St. Andrews, UK

**Keywords:** Golf, Knee, Arthroplasty, Outcomes, Recovery

## Abstract

**Background:**

Approximately 10% to 20% of patients with joint arthroplasties are golfers. The aim of this study was to assess if being a golfer is associated with functional outcomes, satisfaction or improvement in quality of life (QoL) compared to non-golfers following total knee arthroplasty.

**Methods:**

All patients undergoing primary total knee arthroplasty (TKA) over a one-year period at a single institution were included with one-year postoperative outcomes. Patients were retrospectively followed up to assess if they had been golfers at the time of their surgery. Multivariate linear regression analysis was performed to assess the independent association of preoperative golfing status on postoperative function and health-related outcomes.

**Results:**

The study cohort consisted of a total of 514 patients undergoing TKA. This included 223 (43.3%) male patients and 291 (56.7%) female patients, with an overall mean age of 70 (SD 9.5) years. The preoperative Oxford Knee Score (OKS) was significantly higher in golfers when adjusting for confounders (Diff 3.4 [95% CI 1 to 5.8], *p* = 0.006). There was no difference in postoperative outcomes between golfers and non-golfers. There was however a trend towards a higher Forgotten Joint Score (FJS) in the golfers (difference 9.3, 95% CI − 0.2 to 18.8, *p* = 0.056). Of the 48 patients who reported being golfers at the time of their surgery, 43 (89.6%) returned to golf and 88.4% of those were satisfied with their involvement in golf following surgery.

**Conclusions:**

Golfers had better preoperative and equal postoperative knee specific function compared to non-golfers. The majority of golfers returned to golf by one year and were satisfied with their involvement in the game.

**Level of evidence:**

III.

## Introduction

Total knee arthroplasty (TKA) is one of the most cost-effective operative procedures worldwide and is an good intervention for patients suffering from end stage arthritis [28, 29]. Joint arthroplasty (JA) leads to reduce levels of pain and improved levels of function [12, 27]. There are approximately 175,00 hip and knee arthroplasties performed in England, Wales and Scotland each year [11, 17], while there are approximately 1.88 million hip and knee arthroplasties performed in the USA per annum [8]. It has been reported that up to 14% of patients with JA are golfers [14]. Arthritis can have a significant impact on a patients’ quality of life and can prevent golfers from participating in their favoured recreation [19]. Sorbie et al. studied the impact of golf course closure and opening during the COVID-19 pandemic on wellbeing and life satisfaction. They reported that belonging, enjoyment and wellbeing were significantly associated with outdoor course activity and a sense of belonging and satisfaction increased following golf course reopening [21]. It is likely that these findings are applicable to golfers who are unable to play secondary to their arthritis and subsequently return following JA.

TKA in the golfing population has been investigated by Tramer et al, who demonstrated that component type (cruciate-retaining versus posterior-stabilised) has no impact upon pain, performance or stability in those who return to play golf [25]. What is not known, is if being a golfer has any influence on the outcomes compared to other patients undergoing TKA, as their expectations may be different and this has been shown to influence outcome [16, 30]. Furthermore, there is little knowledge of the golfers’ motivation to return to golf following TKA.

The primary aim of this study was to assess if golfers had an equal improvement in their knee specific outcome compared to non-golfers one year following surgery. The secondary aims were to assess (1) preoperative differences in demographics, symptoms and function (2) differences in health-related quality of life (HRQoL), (3) the rates of return to golf at one and five years following surgery, and (4) to assess the influence of golf on motivation and rehabilitation following TKA.

## Patients and methods

Patients were identified from a prospectively compiled arthroplasty database. One year of patients undergoing primary total knee arthroplasty for osteoarthritis were included. All patients received a cruciate-retaining Triathlon (Stryker) prosthesis. Inclusion criteria were given as follows: primary TKR, unilateral surgery, preoperative diagnosis of osteoarthritis, and prospective, preoperative and one-year postoperative outcome measures. Exclusion criteria included those not consenting to follow-up or revision surgery. Demographic and co-morbidity data were collected preoperatively. Patients were retrospectively followed up to assess if they had been golfers at the time of their surgery and questions regarding their involvement and expectations regarding golf postoperatively.

### Surgical protocol

All patients underwent TKA using either a general or spinal anaesthetic. The use of a regional block was at the discretion of the anaesthetist. All surgeries were performed with tourniquets and tranexamic acid was not routinely used. A medial parapatellar approach was used, and intramedullary referencing for the femur and extramedullary referencing for the tibia were employed. Balancing techniques were at the discretion of the surgeon. Knees were performed using mechanical alignment, and the patella was not routinely resurfaced. All patients were mobilised with physiotherapy on day one postoperatively with no restrictions.

### Outcomes measured

The Oxford Knee Score (OKS) [1] was the primary outcome measure and was recorded preoperatively and at 12-month postoperatively. The OKS consists of twelve questions assessed on a Likert scale with values from 0 to 4. A summative score is then calculated where 48 is the best possible score (least symptomatic) and 0 is the worst possible score (most symptomatic). The minimally clinical important difference (MCID) for the OKS is 4.3 points (function) and is thought to represent a clinical difference between two groups of patients [2].

The forgotten joint score (FJS) consists of 12 questions and assesses the awareness of your affected joint during a variety of activities of daily living [36]. Each is scored on a Likert scale ranging from 0 to 4. The total sum of the scores is converted into a scale ranging from 0 to 100, where higher scores reflect less joint awareness during activities of daily living.

The EuroQoL (EQ) general health questionnaire evaluates five domains (5D: assesses mobility, self-care, usual activities, pain/discomfort and anxiety/depression) and was recorded preoperatively and at 12-month postoperatively [6]. The 3L version of the EQ questionnaire was used, with the responses to the five domains being recorded at three levels of severity (no problems, some problems or unable/extreme problems) with 243 possible health states. This index is on a scale of − 0.594 to 1, where 1 represents perfect health, and 0 represents death. Negative values represent a state perceived as worse than death [19]. The second page of the EQ questionnaire consists of a standard vertical 20 cm visual analogue scale (EQ VAS) which is transformed to a scale of 0 (poor health) to 100 (best health) with current health-related quality of life.

The pain visual analogue scale (VAS) is a 15 cm horizontal scale from 0 to 10 where 0 is no pain and 10 is pain as bad as it could be.

Patient satisfaction was assessed by asking the question “How satisfied are you with your operated knee?”. The response was recorded using a five-point Likert scale: very satisfied, satisfied, neither satisfied nor dissatisfied (simplified to neutral for the rest of manuscript), dissatisfied and very dissatisfied. Satisfaction was dichotomised into ‘satisfied’ and ‘dissatisfied’. Satisfied was considered ‘satisfied’ and ‘very satisfied’, and dissatisfied was considered ‘neutral’, ‘dissatisfied’ and ‘very dissatisfied’. Five further questions were asked specifically to those who reported being a golf at the time of the surgery.

### Golf-related outcomes

Golfers were asked if they returned to golf postoperatively and if they were still playing currently. They were also asked if returning to golf was a motivator for undergoing TKA, if they believed golf was beneficial to their recovery and if it improved their overall well-being. Of those patients that returned to golf, they were asked to define how satisfied they were with their involvement in the game of golf since TKA on a five-point Likert scale: very satisfied, satisfied, neutral, dissatisfied and very dissatisfied.

### Statistical analysis

Statistical Package for Social Sciences version 17.0 (SPSS Inc., Chicago, IL, USA) was used for all data analysis. Data were assessed for normality and parametric tests where appropriate. Scalar variables were assessed using either unpaired Student’s t test, or one-way analysis of variance (ANOVA). A Chi square test was used to assess gender, comorbidity and satisfaction differences between groups. Fisher’s exact test was used for groups < 5. Significance was set as a *p*value of < 0.05. Multivariate linear regression analysis was performed to assess for golfing status as a preoperative independent variable when adjusted for preoperative confounders. Binary logistic regression was also performed to assess if golfing status predicted postoperative satisfaction when adjusting for confounders.

A power calculation was performed using the MCID for the OKS (primary outcome measure) of 4.3, a standard deviation of 10 points (effect size 0.43), with an alpha of 0.05 and two tailed analysis with 48 in the golfing group and 466 in the non-golfing group achieved 81% power.

### Ethics

Ethical approval was obtained from the regional ethics committee (Research Ethics Committee, South East Scotland Research Ethics Service, Scotland [16/SS/0026]) for analysis and publication of the presented data. The data collection was carried out in accordance with the GMC guidelines for good clinical practice and the Declaration of Helsinki.

## Results

### Study cohort characteristics

The study cohort consisted of a total of 514 patients undergoing TKR with complete preoperative and one-year postoperative data that met the inclusion criteria. This included 223 (43.3%) male patients and 291 (56.7%) female patients, with an overall mean age of 70 (SD 9.5) years and a mean BMI of 30.1 (SD 5.9). Two hundred and seventy-six TKA were left (53.7%), and 238 (46.3%) were right sided. Thirty-six patients had died at the time of golfing status assessment (i.e. five years postoperatively). All identified golfers played right-handed. Preoperative demographic comparisons between the golfer cohort (*n* = 48) and the non-golfer cohort (*n* = 466) can be seen in Table 1.

**Table 1:** Preoperative demographics and functional outcomes between both golfers and non-golfers

Demographic	Golfer			Difference/odds ratio (95% CI)	*p* value
	Study cohort (*n* = 514)	No (*n* = 466)	Yes (*n* = 48)		
Gender (%)					
Male	223 (43.3)	181 (37.2)	42 (87.5)		
Female	291 (56.7)	285 (62.8)	6 (12.5)		< 0.001
Side (%)					
Left	276 (53.7)	252 (54.1)	24 (50)		
Right	238 (46.3)	214 (45.9)	24 (50)	OR 1.2 (0.6 to 2.1)	0.59
Mean age (years, SD)	70 (9.5)	70.2 (9.6)	68.5 (8.2)	Diff − 1.7 (− 4.5 to 1.2)	0.25
BMI	30.1 (5.9)	30.6 (5.9)	29.7(5.9)	Diff − 0.9 (− 2.6 to 0.9)	0.33
Co-morbidities (*n*, %)					
IHD	25	22	3	OR 1.4 (0.4 to 4.7)	0.64
COPD	19	18	1	OR 0.5 ( 0.1 to 4)	0.446
Vascular disease	7	6	1	OR 0.7 0 (0.4 to 1.3)	0.81
Diabetes	57	50	7	OR 1.4 (0.6 to 3.1)	0.64
Gastric ulcer	10	10	0	OR 1 (1 to 1)	0.72
Kidney disease	9	8	1	OR 1.2 (0.2 to 9.7)	0.74
Liver disease	4	4	0	OR 1 (1 to 1)	0.92
Cerebrovascular disease	16	14	2	OR 0.7 (0.1 to 5.8)	0.98
Preoperative EQ5D VAS	70.8 (18.8)	70.1 (19.1)	77.1 (14.7)	Diff 7 (1.5 to 12.6)	0.01
Preoperative EQ5D Index	0.423 (0.312)	0.414 (0.31)	0.449 (0.318)	Diff 0.084 (− 0.001 to 0.177)	0.07
Preoperative Pain VAS	51.6 (21.8)	51.3 (21.6)	55.1 (25.5)	Diff 3.8 (− 2.7 to 10.3)	0.25
Preoperative OKS	20.9 (8)	20.4 (7.9)	25.3 (7.8)	Diff 4.9 (2.5 to 7.2)	< 0.001

*BMI* Body mass index, *SD* Standard deviation, *IHD* Ischaemic heart disease, *COPD* Chronic obstructive pulmonary disease, *EQ5D* EuroQol 5 dimension, *VAS* Visual analogue scale, *OKS* Oxford knee score

### Functional and health-related outcomes

There was no difference in postoperative functional or health-related outcomes between golfers and non-golfers (Table 2) which persisted when adjusting for preoperative confounders (Table 3). Preoperative OKS was significantly higher in golfers compared to non-golfers and when adjusted for sex differences between the groups golfing status was independently associated with greater preoperative OKS score (difference 3.4 [95% CI 1 to 5.8], *p* = 0.006). HRQoL was also greater in golfers which was significant for EQ5D VAS with a trend towards significance in EQ5D Index. There was also a trend towards significance (*p* = 0.056) for golfers to be less aware (FJS) of their knee joint postoperatively compared to non-golfers (Table 2).

**Table 2:** Comparison of postoperative variables between golfers and non-golfers

Golfer			Difference/odds ratio (95% CI)	*p* value
Demographic	No (*n* = 466)	Yes (*n* = 48)		
EQ5D VAS				
1 year	77.6 (18.5)	81.5 (77.6)	3.8 (− 1.6 − 9.4)	0.166
Change	7.4 (19.6)	4.4 (17.6)	− 3.1 (− 8.9 − 2.7)	0.298
EQ5D Index				
1 year	0.74 (0.268)	0.773 (0.262)	0.03 (− 0.05 − 0.1)	0.408
Change	0.325 (0.334)	0.274 (0.346)	− 0.051 (− 0.151 − 0.49)	0.314
Pain VAS				
1 year	72.9 (26.9)	74.8 (28.4)	4.1 (− 6.2 − 9.9)	0.657
Change	21.5 (31.3)	19.7 (35.6)	4.82 (− 11.2 − 7.7)	0.715
OKS				
1 year	35.5 (10)	38.1 (10.6)	2.6 (− 0.4 − 5.6)	0.089
Change	15 (9.8)	12.7 (11.4)	− 2.4 (− 5.4 − 1.1)	0.118
FJS				
1 year	50.1 (31.8)	59.4 (33.1)	9.3 (− 0.2 − 18.8)	0.056

*EQ5D* EuroQol 5 dimension, *VAS* Visual analogue scale, *OKS* Oxford knee score, *FJS* Forgotten joint score

**Table 3:** Multivariate linear regression analysis of golfing status post-operatively as an independent predictor when adjusting for significant preoperative variables (*p* < 0.1) to the model (Gender, preoperative EQ-5D index, preoperative OKS)

Outcome measure	B	95% CI		*p* value
EQ5D Index (*r*^2^ = 0.2)				
Non-golfer	Reference			
Golfer	− 0.01	− 0.1	0.7	0.765
Pain VAS (*r*^2^ = 0.1)				
Non-golfer	Reference			
Golfer	− 0.4	− 8.7	7.9	0.92
OKS (*r*^2^ = 0.2)				
Non-golfer	Reference			
Golfer	0.05	− 2.9	3	0.97
FJS (*r*^2^ = 0.1)				
Non-golfer	Reference			
Golfer	2.7	− 7	12.3	0.59

*EQ5D* EuroQol 5 dimension, *VAS* Visual analogue scale, *OKS* Oxford knee score

At one-year follow-up, there were 35 golfers (74.5%) who were satisfied and 12 (25.5%) who were dissatisfied, compared to 367 non-golfers (80.8%) who were satisfied and 87 (19.2%) who were dissatisfied (*p* = 0.297). Following binary regression analysis adjusting for preoperative confounders, there was no difference in satisfaction between golfers and non-golfers (*p* = 0.22).

### Returning to golf following TKA

Of the 48 patients who actively participated in golf at the time of their surgery, 43 (89.6%) returned to golf within 1 year and 31 (65%) were still playing five years postoperatively. Of those that were no longer playing, two patients associated this with problems related to their TKA. Thirty patients (62.5%) reported golf as being an important reason for undergoing surgery. Thirty-two patients (66.7%) reported that they felt golf helped with their rehabilitation and 39 (81.3%) felt returning to golf improved their overall well-being. Of those that returned to golf, thirty-eight patients (88.4%) were deemed to be satisfied overall with their involvement in golf following surgery. There was no difference in health-related or functional outcomes in golfers when comparing left and right-sided surgery at one year (*p* > 0.41). There were no surgical complications in the golfer cohort.

## Discussion

This study has shown that golfers had an outcome that was equal to non-golfers according to knee-specific function, HRQoL and satisfaction. However, they did have higher preoperative knee-specific function and HRQoL scores. The rate of return of golfers was high with ninety per cent of golfers returning at one year postoperatively, with a self-reported satisfactory involvement in the game of golf achieved by 88% of golfers undergoing TKA.

Golf can provide moderate intensity physical activity (with a reported general metabolic equivalents [MET] of 4.8)[9], while one ‘round’ of 18 holes can burn approximately 1200kcals and players will perform 11,000 to 16,000 steps over a distance of 4–8 miles [7, 23]. Some of these health benefits may explain why golfers were found to have superior functional preoperative scores compared to the rest of a population undergoing JA. However, it was interesting to note that there was no difference in BMI between the two cohorts. Golfers also reported better HRQoL compared to non-golfers. A previous Swedish study of 300,818 golfers and non-golfers reported a 40% lower mortality rate in the golfing cohort, which correlated to a 5-year increase in life expectancy regardless of gender, age or socioeconomic status [4].

The overall prevalence of golfers was 9%, which increased to 23% for male patients. This may be unique to the demographics of the population assessed; however, a previous UK study has reported approximately 14% of hip arthroplasty patients were golfers in their cohort [14]. In the current study, 89.6% of the golfers returned to golf following TKA surgery. A recent meta-analysis has reported a 70% rate of returning to golf after TKA [15]. Motivation for returning to golf after JA and patients’ satisfaction with their golfing involvement has not previously been investigated. Golfers in this study reported that getting back to playing sport contributed to their reason for undergoing joint replacement in 63% of cases. Clinicians should be aware of this when counselling patients regarding TKA and the likelihood of being able to return. It was reassuring to report that of those that returned, 88% were satisfied with their involvement in the game of golf after surgery. Arguably, these data are more important than exploring postoperative performance-related metrics such as changes in handicap or driving distance, which are influenced by other factors such as increasing age [22]. The process of playing golf requires significant balance [26], muscular function [10] and strength [20] and returning to the sport may be a surrogate marker for patients coping with their new joint replacement.

The physical and mental health benefits of golf were reported by our cohort of golfers with two thirds believing golf contributed to their rehabilitation and 81% believing returning to the game improved their overall well-being. This is in keeping with a previous study during the COVID-19 pandemic which showed that sense of belonging, enjoyment and well-being were significantly associated with returning to play golf after activity restrictions were lifted following lockdown [21]. Despite golfers having superior preoperative scores compared to non-golfers, there was no difference in functional outcomes between the two groups postoperatively. Although we cannot conclude why this is, it may be that golfers’ who have lower thresholds were undergoing TKA due to arthritis limiting their hobby or that they have greater expectations postoperatively compared to non-golfers.

### Limitations

This study must be interpreted in light of its limitations. There was a predominance of males in the golfing cohort. This is however reflective of the overall golfing community, and previous studies have shown no influence on gender following TKA [3, 13, 24]. Furthermore, we did adjust for gender during the regression analysis. The severity or pattern of osteoarthritis within the knee prior to surgery was not assessed, and this may contribute to attaining satisfaction and acceptable patient outcomes postoperatively although there is limited evidence to support this [18]. In addition, this is a UK-based study where golf is a popular sport. The results therefore may not be applicable in other countries where golf is less prevalent.

## Conclusion

Golfers have better knee specific function prior to TKA compared to non-golfers. Postoperative functional outcomes are equal between both groups. The majority of golfers will return to golf by one year and be satisfied with their involvement in the game.

## Data Availability

The datasets used and/or analysed during the current study are available from the corresponding author on reasonable request.

## Code availability

Statistical Package for Social Sciences (SPSS) software (IBM, Inc., Armonk, New York, United States) v24.
